# Massive Central Pulmonary Embolism with Riding Embolus and Concomitant Aortic Arch Embolism—Should We Diagnose Patients Earlier for Blood Clotting Disorders? Case Report

**DOI:** 10.3390/jcdd12010026

**Published:** 2025-01-14

**Authors:** Anna Lis, Paweł Kowalski, Marcin Wita, Tomasz Zawadzki, Tomasz Ilczak, Wojciech Żurawiński, Mateusz Majewski

**Affiliations:** 1 Emergency Department, Leszek Giec Upper-Silesian Medical Centre of the Medical University of Silesia in Katowice, 40-635 Katowice, Poland; 2 I Department of Cardiology, Leszek Giec Upper-Silesian Medical Centre of the Medical University of Silesia in Katowice, 40-635 Katowice, Poland; 3 Department of Emergency Medicine, Faculty of Health Sciences, University of Bielsko-Biala, 43-309 Bielsko-Biała, Poland; 4 Department of Emergency Medicine, Faculty of Medical Sciences in Katowice, Medical University of Silesia in Katowice, 40-635 Katowice, Poland

**Keywords:** pulmonary embolism, aortic arch embolism, paradoxical embolism, hypercoagulability, antithrombin III deficiency, ischaemic stroke

## Abstract

Paradoxical embolism occurs when a clot originates in the venous system and traverses through a pulmonary or intracardiac shunt into the systemic circulation, with a mortality rate of around 18%. The risk factors for arterial embolism and venous thrombosis are similar, but different disease entities can lead to a hypercoagulable state of the blood, including antithrombin III (AT III) deficiency. We report the case of a 43-year-old man with a massive central pulmonary embolism with a rider embolus and concomitant aortic arch embolism with involvement of the brachiocephalic trunk, bilateral subclavian and axillary arteries, and the right vertebral artery, followed by a secondary ischaemic stroke. The Pulmonary Embolism Response Team (PERT) consulted the patient on several occasions; he was treated initially with an intravenous infusion of unfractionated heparin under activation partial thromboplastin time (APTT) and AT III substitution. After several days of hospitalisation and the conversion of pharmacotherapy to oral anticoagulants, the patient was discharged home in a stable condition with recommendations for further follow-up in appropriate clinics. This case highlights the role of in-depth diagnostics for coagulation disorders in patients after pulmonary embolism, especially without known risk factors.

## 1. Introduction

Hypercoagulability refers to an increased tendency of clotting, as a result of exaggerated coagulation. Arterial embolism, like myocardial infarction or stroke, differs from venous thromboembolism (VTE), represented by two main events: deep vein thrombosis (DVT) and pulmonary embolism (PE), in terms of pathophysiology and treatment; however, both share some risk factors [1,2,3]. Paradoxical embolism is a phenomenon whereby the clot originates in the venous system and traverses through a pulmonary or intracardiac shunt into the systemic circulation, with a mortality rate of around 18% [4,5]. Various diseases can lead to a hypercoagulable state, such as the antithrombin III (ATIII) deficiency discovered in 1965 by Egeberg et al. [1,6]. Other coagulation disorders include protein C deficiency, prothrombin G20210A mutation, hyperhomocysteinemia, and antiphospholipid syndrome (APS). Malignancy leads to a prothrombotic state through the production of procoagulant factors and is the second most common acquired hypercoagulability [1]. The case we describe raises the question of the careful diagnosis of blood clotting disorders after a first incident of PE with no detectable risk factors; however, in-depth diagnosis depends on various aspects like the type of VTE or the age of first presentation.

## 2. Case Report

A 43-year-old patient was admitted to the emergency department (ED) due to pain, numbness, and paresis of the upper limbs for approximately 5 h. He had a history of unprovoked PE one year earlier, continued therapy with rivaroxaban for 6 months, and then the drug was discontinued due to a first incident of PE and, as the patient stated, no further tests were performed, except for the confirmation of DVT after PE incident; however, there was no documentation available. Past medical history involved schizophrenia treated for 4 years.

**Figure 1:**
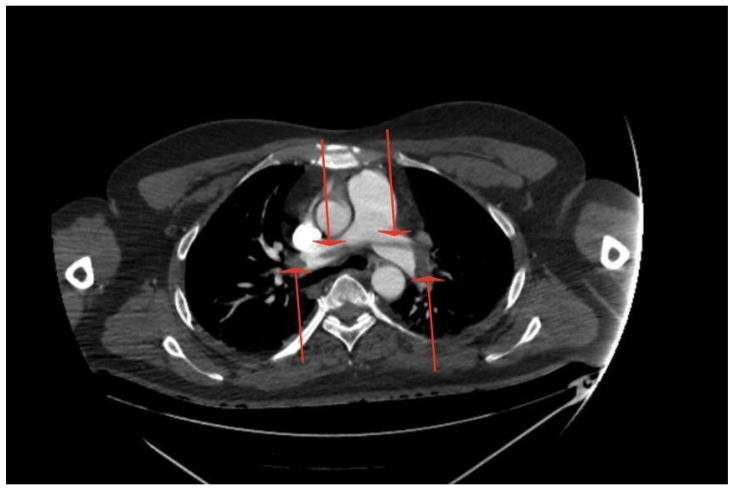
Massive central pulmonary embolism with riding embolus (red arrows).

**Figure 2:**
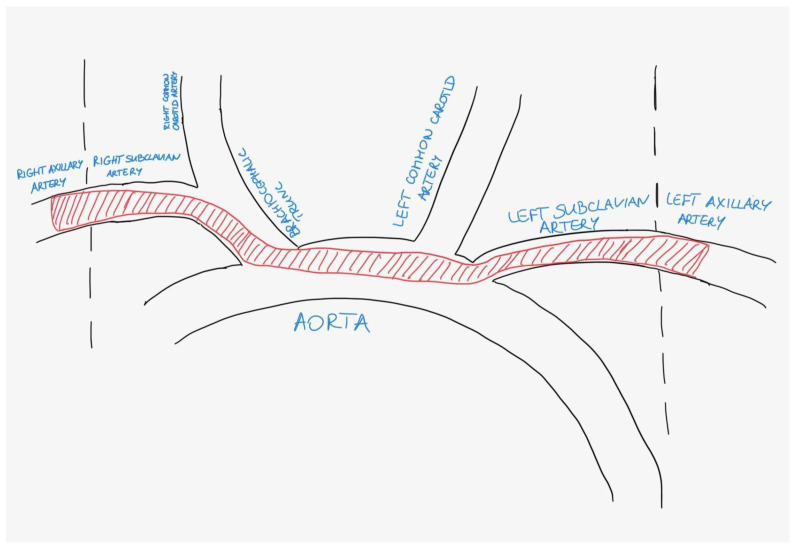
Scheme of the embolism (red colour) of the aortic arch and its branches.

**Figure 3:**
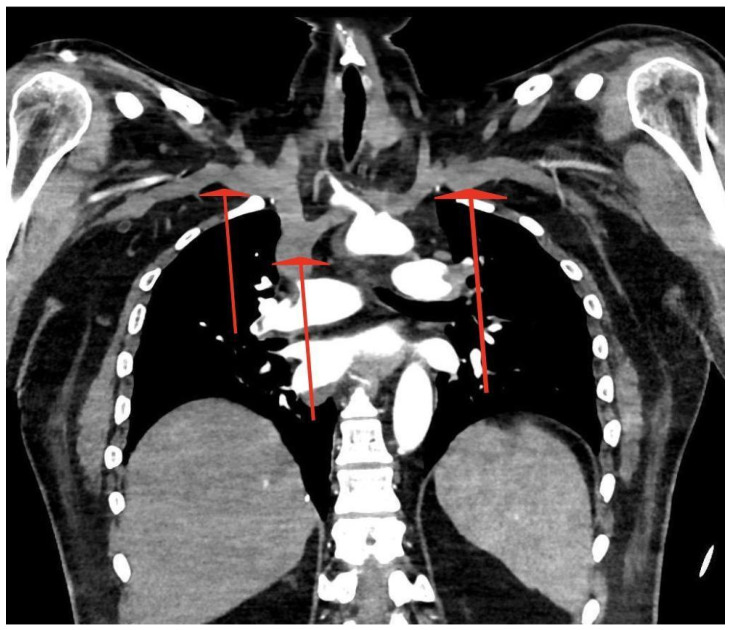
Embolism (red arrows) of the aortic arch with the involvement of the brachiocephalic trunk, bilateral subclavian and axillary arteries, and the right vertebral artery.

On admission, the patient had an intact Glasgow Coma Scale (GCS 15) despite looking cyanotic on the face. There were no signs of trauma, but blood pressure (BP) and saturation (SpO_2_) were unable to be obtained from the upper limbs. Although the capillary refill time (CRT) was >5 s bilaterally, both upper limbs were cold and there were no palpable pulses on either radial artery. The blood pressure and saturation, as measured on the lower extremities, showed BP 130/70 mmHg, heart rate (HR) 80/min, and SpO_2_ 98% without oxygen supplementation. The blood gas analysis is shown in Table 1: as the initial sample was obtained from the vein, the only value that could be reliably assessed was the elevated level of lactate. Due to the suspicion of aortic dissection, angio-CTs of the thoracic and abdominal aorta, as well as pulmonary arteries, were immediately performed, revealing massive central PE with riding embolus: embolic material started at the division of the pulmonary trunk with the greatest accumulation at the division of the pulmonary arteries into the lobar arteries. In addition, embolic material was present in numerous segmental arteries and a few subsegmental arteries of both pulmonary arteries in all lobes. At the same time, embolism of the aortic arch with involvement of the brachiocephalic trunk, bilateral subclavian, and axillary arteries and the right vertebral artery was visualised; suspicion of a patent foramen ovale (PFO) was raised [Figure 1, Figure 2 and Figure 3]. No source of emboli in the caval system was found.

On the return to the ED, a cardiac surgeon, vascular surgeon, and invasive cardiologist were called and transthoracic echocardiography (TTE) of the heart was requested. A Pulmonary Embolism Response Team (PERT) consultation took place. The treatment was initiated: unfractionated heparin (UFH) bolus of 5000 units intravenously and 25,000 units by intravenous infusion for 24 h. Due to severe elbow pain, intravenous morphine was administered. TTE showed features of right ventricular (RV) overload: D-sign—a shift of the interventricular septum towards the left side of the heart and positive McConell’s sign—akinesis of the mid free RV-wall, and normal motion of the apex; the left ventricle (LV) was compressed by the RV; a 28 [mm] diameter aortic arch was measured with apparent flow limitation; no atrial septal leak was seen. Control arterial blood gas (ABG) taken after 1.5 h showed a mild respiratory acidosis, with slightly elevated lactate level [Table 1]. Important laboratory findings are presented in Table 2.

Various options were considered within the PERT—it was decided that only the UFH infusion would be kept, as mechanical thrombectomy or the administration of alteplase carried too high a risk of complications. Vital signs remained within normal range with BP measured on the lower extremity. After an hour, TTE showed reduced features of RV overload. The mobility and sensation of the upper limbs improved, but they remained pale and cold. The peripheral cyanosis disappeared, but the pulse on the radial arteries was still absent. The patient was transferred to the Intensive Care Unit and then to the Cardiology Department for further treatment.

During this time, UFH infusion was continued according to the activated partial thromboplastin time (APTT) levels, and acetylsalicylic acid (ASA), fluids, and electrolyte therapy were administered. ATIII activity was slightly reduced: 72.82[%] with reference interval 79.40–112.00; therefore, 1000 units of ATIII were transfused. However, during the following days, ATIII activity decreased. An angio-CT scan after two days showed regression of the embolus material in the pulmonary trunk division. The images of other arteries were comparable. Repeated head CTs were performed, showing an ischaemic stroke secondary to massive embolism. Surgical/vascular, cardiothoracic, and psychiatric consultations took place, and the maintenance of the pharmacotherapy, including the treatment of schizophrenia with olanzapine, was recommended—despite the increased risk of thromboembolism—because of the threat of acute psychosis on withdrawal. CA 19-9 and CEA markers came back normal. Between days 3 and 6, the patient remained in critical condition, dependent on an intravenous infusion of dopamine and norepinephrine, which were slowly deescalated under BP control. On day 9, the pulse was present on the right radial artery, and the UFH infusion was changed to subcutaneous injections of low-molecular-weight heparin (LMWH). A Doppler examination of the arteries of the neck showed bilateral cephalad flow in the carotid and vertebral arteries, with a normal spectrum. An ultrasound examination of the lower extremities’ veins showed thrombosis in the left femoral vein at the central line insertion site; otherwise, no thrombotic findings were observed. On day 11, an angio-CT scan showed the regression of embolic material from the aortic arch, brachiocephalic trunk, right subclavian artery, proximal segment of the right common carotid artery, and both axillary arteries and the significant regression of embolic material from the left subclavian artery. Transoesophageal echocardiography (TEE) excluded a PFO. However, a large interatrial septal (IAS) aneurysm was incidentally identified; features of leakage or thrombus in IAS or cavities in a colour Doppler and after contrast injection with Valsalva test were not visualised. The patient was consulted neurologically to determine secondary prevention of stroke—a switch to a direct oral anticoagulant (DOAC), rivaroxaban, was planned for 3 weeks at a dose of 2 × 15 [mg], and then 1 × 20 [mg] permanently with a reduction in ASA dosage to 1 × 75 [mg] and the addition of a statin. Tests for antiphospholipid syndrome and systemic vasculitis came back negative. A Holter electrocardiogram recorded no pathologies. On day 13, the patient was discharged home in a stable condition with recommendations for further treatment under the supervision of the hypercoagulability, cardiology, and neurology clinics. During the hospital stay, the source of the embolic material was not found; the patient was referred for further outpatient care.

## 3. Discussion

According to the European Society of Cardiology (ESC) guidelines for the diagnosis and management of acute PE, the immediate initiation of anticoagulant treatment with weight-adjusted LMWH or fondaparinux is preferred. UFH is only considered for unstable patients or for those with a high risk of bleeding; similar recommendations are given in the update based on the revised Association of the Scientific Medical Societies in Germany (AWMF) S2k Guideline on the management of DVT [7,8]. The patient we described did not show signs of shock on admission to the ED—he maintained normal BP values without the need for catecholamines, and his HR and SpO_2_ remained normal. However, together with PERT specialists, we decided to start with UFH treatment as, in the beginning, it was still unclear whether the surgical approach would be pursued, and the effect of UFH could be quickly reversed. The Pulmonary Embolism Thrombolysis (PEITHO) trial showed that, among normotensive patients and intermediate-risk PE, defined as the presence of RV cardiac dysfunction and elevated troponin levels, thrombolysis was associated with a significant reduction in the risk of haemodynamic decompensation or shock, but at the same time with an increased risk of major extracranial and intracranial bleeding [9]. Other options considered were pulmonary trunk mechanical thrombectomy up to open surgery, which involved putting the patient into deep hypothermia and connecting extracorporeal blood oxygenation (ECMO), followed by the removal of embolic material from both the pulmonary arteries and aorta. Studies comparing survival and PE recurrence rates among patients treated with thrombolysis or with embolectomy as first-line therapy showed no difference in terms of 30-day mortality or 5-year survival, but thrombolysis was associated with a higher risk of stroke, recurrent PE, and re-intervention after 30 days [10]. Aymard T. et al. showed that surgical embolectomy is an excellent treatment option for massive PE with comparable early mortality and significantly fewer bleeding complications than thrombolysis [11]. In the case described, embolic material was present not only in the pulmonary vessels, but also in the aortic arch and its branches. Furthermore, with the symptoms of RV overload present, the connection of ECMO was associated with their significant exacerbation and higher risk of death. The removal of embolic material from the arteries peripherally, or systemic thrombolysis, was also considered, but this was associated with a high risk of thrombus fragmentation and the displacement of embolic material into the cephalic arteries, which would result in ischaemic stroke. As Herman C.R. et al. described, aortic arch repair itself carries a significant risk of stroke: 4.7–6.0%. The endovascular approach lowers the risk, as it avoids extracorporeal circulation and possible hypothermic circulatory arrest; however, strokes after endovascular procedures of the aortic arch still show a significant risk rate of 4.2–5.9% [12]. Ultimately, it was therefore decided to retain only intravenous UFH infusion under APTT control, as the other options carried too much risk, and the patient was regularly reassessed by the PERT specialists.

In our patient, the first episode of PE occurred at a young age, with no prior history of fracture, immobilisation, or nicotinism; DVT was diagnosed later and no further investigation for coagulation disorders was pursued. The causes of the aortic embolism detected during the second PE incident remain unknown; perhaps the embolic material originated in the cardiac cavities and then migrated into the aorta, but it is usually associated with the presence of a PFO, which was excluded in the case described [13,14]. It is crucial to try to understand the origin of the embolus, principally in the aortic arch, as it is not possible that it formed in that location—further diagnostics like nuclear magnetic resonance (NMR) of the heart could provide additional information. Paradoxical embolism diagnosis requires confirmation of a venous source of embolism, an endocardial defect, or pulmonary fistula, and evidence of arterial embolism [14,15,16]. However, in the case described here, no PFO was demonstrated by TEE, which strongly suggests the need for an in-depth diagnostic workup for blood diseases and genetic testing in young patients after the first episode of PE with no detectable risk factors. Patients with confirmed antithrombin, protein C, or protein S deficiency are often candidates for indefinite anticoagulant treatment after a first episode of PE occurring in the absence of a major reversible risk factor and the testing for thrombophilia may be considered, especially when occurring against the family history of PE [7,17,18]. Studies conducted after the incident described above demonstrated mild ATIII deficiency—an antithrombin level of 73% at diagnosis is not necessarily a sign of hereditary antithrombin deficiency, but it could happen due to the acute thrombosis or heparin infusion; follow-up measurements would be required. ATIII binds to heparin on endothelial cells and forms a complex with thrombin, thus inhibiting coagulation. The condition manifests as thrombosis under 50 years of age and it is associated with the highest risk of thrombotic events among hereditary thrombophilias [1,19]. ATIII can be substituted, and according to ESC guidelines, patients with ATIII deficiency are candidates for indefinite anticoagulation treatment [7]. This patient would be treated with indefinite anticoagulation regardless of that due to recurrent unprovoked VTE.

## 4. Conclusions

Thromboembolic diseases are one of the leading causes of mortality worldwide. The case described here highlights the role of in-depth diagnosis for coagulation disorders in patients after PE, especially at a young age and without known risk factors. In this case, the diagnosis was made during the second incident of PE, demonstrating AT III deficiency; however, the cause of the aortic embolism remains unknown. In the event of massive central PE with riding embolus and concomitant aortic arch embolism with involvement of the brachiocephalic trunk, bilateral subclavian and axillary arteries, and right vertebral artery, it was decided that treatment with UFH infusion would be pursued under APTT control and AT III substitution, in this case achieving an improvement in the patient’s clinical condition; however it is crucial to assess such complex cases individually and make therapeutic decisions together with PERT specialists. Given the second episode of PE, the AT III deficiency found and the history of ischaemic stroke secondary to massive embolism, the decision was made to initiate treatment with DOACs indefinitely, while reducing the dose of ASA and adding a statin as secondary prevention of stroke.

## Figures and Tables

**Table 1:** Blood gasometry comparison.

Parameter	Venous Blood Gasometry on Admission to the ED ^1^	ABG ^1^ After 1.5 h	ABG ^1^ Day 5
pH N ^1^: 7.35–7.45	7.368	7.327	7.411
pCO_2_ ^1^ [mmHg] N ^1^: 32.0–48.0	49.4	51.0	45
pO_2_ ^1^ [mmHg] N ^1^: 83–108	41.1	136	63
lactate [mmol/L] N ^1^: 0.3–0.8	2	1.8	normal
BE ^1^ [mmol/L]	3.1	0.7	

^1^ ED—emergency department; ABG—arterial blood gas; BE—base excess; N—normal range; pCO_2_—partial pressure of carbon dioxide; pO_2_—partial pressure of oxygen.

**Table 2:** Laboratory parameters.

Parameter	Value at Admission	Day 1	Day 4	Day 7	Day 10
D-dimer [ng/mL] N ^1^: 0–550	12,077.94		6285.66	5328.92	5518.85
AT III [%] ^1^ N ^1^: 79.40–112.00		72.82	68.80	62.39	
Fibrinogen [g/L] N ^1^: 1.80–3.50			6.58	6.51	
hs TnT [ng/mL] ^1^ N ^1^: <0.014	0.039				
CRP [mg/L] ^1^ N ^1^: <5.0	12.5		43.5	12.1	
APTT [s] ^1^	28.7				
PT ^1^ [s] N ^1^: 9.4–13.4	11.5				
INR ^1^ N ^1^: 0.88–1.20	1.0				

^1^ N—normal range (laboratory’s reference intervals); AT III—antithrombin III; hs TnT—high-sensitivity troponin T; CRP—C-reactive protein; APTT—activated partial thromboplastin time; PT—prothrombin time; INR—international normalized ratio.

## Data Availability

Data are contained within the article.
